# Targeting Adenylate Cyclase Family: New Concept of Targeted Cancer Therapy

**DOI:** 10.3389/fonc.2022.829212

**Published:** 2022-06-27

**Authors:** Rui Guo, Tian Liu, Marzieh Dehghan Shasaltaneh, Xuan Wang, Saber Imani, QingLian Wen

**Affiliations:** ^1^ Department of Oncology, The Affiliated Hospital of Southwest Medical University, Luzhou, China; ^2^ Department of Biology, Faculty of Science, University of Zanjan, Zanjan, Iran; ^3^ China Regional Research Center, International Centre for Genetic Engineering and Biotechnology Taizhou, Jiangsu, China

**Keywords:** adenylate cyclase, cAMP signaling, molecular targeted therapy, chemoresistance, signaling pathway 4

## Abstract

The adenylate cyclase (ADCY) superfamily is a group of glycoproteins regulating intracellular signaling. ADCYs act as key regulators in the cyclic adenosine monophosphate (cAMP) signaling pathway and are related to cell sensitivity to chemotherapy and ionizing radiation. Many members of the superfamily are detectable in most chemoresistance cases despite the complexity and unknownness of the specific mechanism underlying the role of ADCYs in the proliferation and invasion of cancer cells. The overactivation of ADCY, as well as its upstream and downstream regulators, is implicated as a major potential target of novel anticancer therapies and markers of exceptional responders to chemotherapy. The present review focuses on the oncogenic functions of the ADCY family and emphasizes the possibility of the mediating roles of deleterious nonsynonymous single nucleotide polymorphisms (nsSNPs) in ADCY as a prognostic therapeutic target in modulating resistance to chemotherapy and immunotherapy. It assesses the mediating roles of ADCY and its counterparts as stress regulators in reprogramming cancer cell metabolism and the tumor microenvironment. Additionally, the well-evaluated inhibitors of ADCY-related signaling, which are under clinical investigation, are highlighted. A better understanding of ADCY-induced signaling and deleterious nsSNPs (p.E1003K and p.R1116C) in ADCY6 provides new opportunities for developing novel therapeutic strategies in personalized oncology and new approaches to enhance chemoimmunotherapy efficacy in treating various cancers.

## 1 Introduction

The latest global cancer burden data released by the International Agency for Research on Cancer (IARC) of the World Health Organization revealed 19.29 million new cancer cases and 9.96 million cancer deaths worldwide in 2020. The number of new cancer cases is predicted to increase to approximately 28.4 million worldwide in 2040, which is up by 47% compared to 2020. Following the development of targeted therapies and immunotherapy, cancer treatment modalities have long gone beyond surgery and radiotherapy. Outcomes for cancer patients are improved by applying tyrosine kinase, cell cycle-dependent kinase (CDK), cell cycle, integrin signaling, and immune checkpoint inhibitors, although their efficacy significantly varies due to a variety of limiting factors (1). The effectiveness of targeted therapy depends on the correct matching between drug and target. The targets are expressed to varying degrees on tumor cells and act as the oncogenic driver mutations that provide cancer cells with growth and survival advantages. Therefore, the specific mechanisms of tumorigenesis should be further explored to identify novel therapeutic targets meeting the challenge related to the precision of oncology.

Based on the results of many studies since 1971, adenylate cyclase (AC) activity and cAMP levels are associated with the normalization of tumor cell morphology and restoration of contact inhibition, as well as the reduced growth rate (2, 3). AC activation, which drives the production of cAMP from adenosine triphosphate (ATP), leading to elevated intracellular cAMP levels (4), is accompanied by cancer through protein kinase A (PKA)-dependent and independent pathways. In addition to being involved in cell cycle inhibition, apoptosis, and growth, cAMP regulates angiogenesis by inhibiting vascular endothelial growth factor (VEGF), TGF-β, and epidermal growth factor receptor (EGFR) pathways (5). Targeting the upstream and downstream of the cAMP signaling pathway exhibits a range of anticancer effects such as the induction of mesenchymal-to-epithelial transition (EMT), inhibition of cell growth and migration, and enhanced sensitivity of cancer cells to conventional anticancer drugs (6–8).

The *adenylate cyclase* (*ADCY*) gene family, which encodes AC proteins, plays important regulatory roles in a spectrum of biological processes like cell proliferation, apoptosis, migration, invasion, angiogenesis, abnormal metabolism, and immune escape (9–11). AC compounds are already in clinical use for certain diseases such as neuropathic pain, neurodegenerative diseases, congestive heart failure, asthma, and male contraception (12). However, the studies on the role of the various isoforms of the ADCY family in cancer have been mainly limited to the screening of biomarkers for different cancers and the discovery of interactions with other genes or small molecules (13). Certain AC isoforms are expressed more or less in specific cancers and are associated in tumor development as pro- or anticancer factors, although specific mechanisms are unclear. In general, the potential role of the family in cancer therapy warrants attention and can yield important new targets for the treatment. The present study reviewed the current understanding of the ADCY family and the mechanism of ADCY activation in response to various types of chemotherapy. It highlighted the role of the family in the different signaling pathways regulating resistance to chemotherapy in tumorigenesis.

## 2 ACs and cAMP Level Regulation

**Figure 1:**
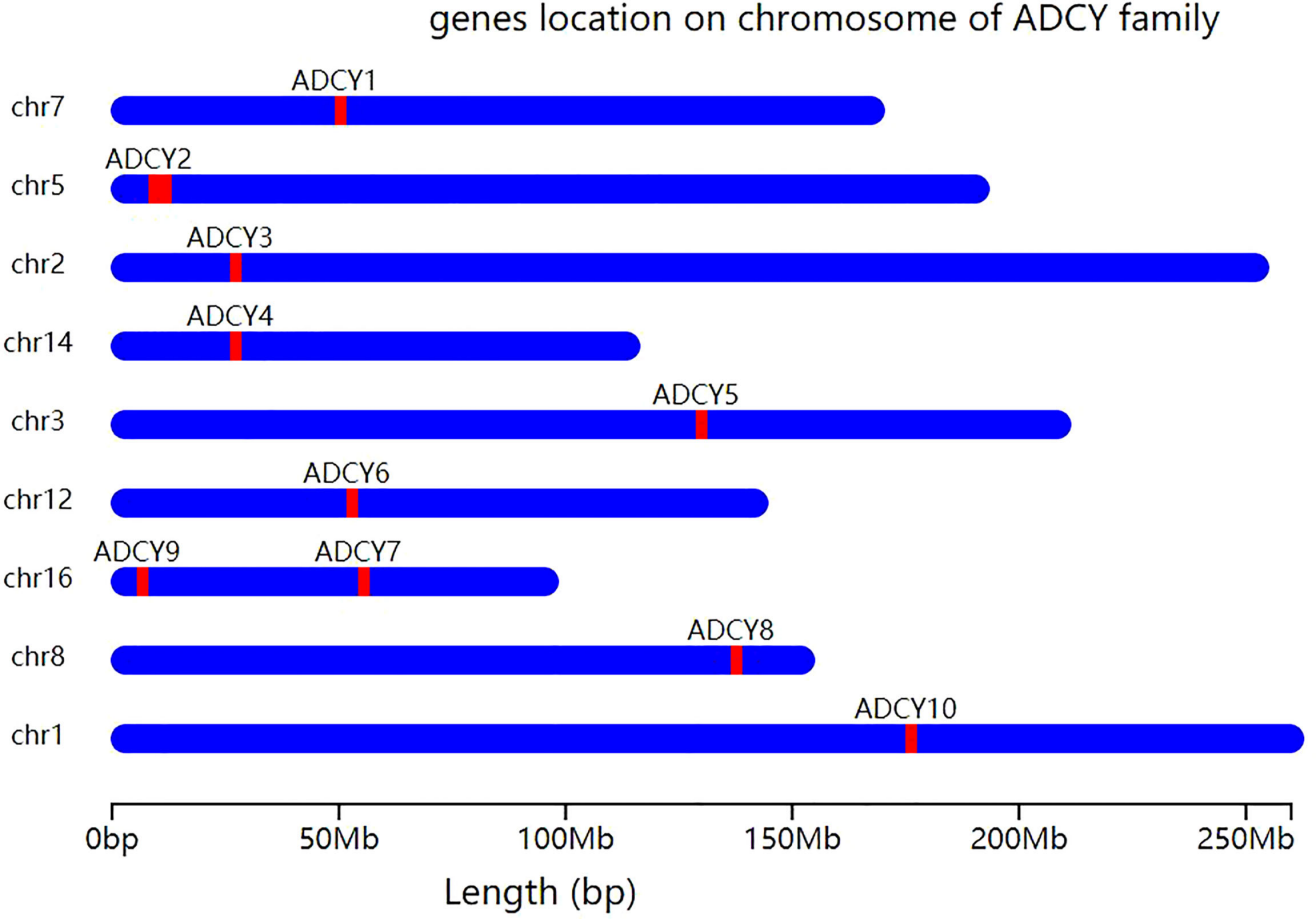
Chromosomal localization of *ADCY* gene isoforms. Except for *ADCY7* and *ADCY9*, which are both located on chromosome 16, all other *ADCY* subtypes are located on different chromosomes. *ADCY1* is located on chromosome 7, *ADCY2* on chromosome 5, *ADCY3* on chromosome 2, *ADCY4* on chromosome 14, *ADCY5* on chromosome 3, *ADCY6* on chromosome 12, *ADCY8* on chromosome 8, and *ADCY10* on chromosome 1.

ACs are glycoproteins with important functions as intracellular signaling regulators. This large family contains 10 subtypes (AC I–IX and soluble AC (sAC)), each of which is encoded by an independent gene (*ADCY1*–*ADCY10*). *ADCY1–ADCY18* are located on different chromosomes, while *ADCY9* and *ADCY7* are locateded on chromosome 16 (14) (**Figure 1**). Membrane-bound ACs can be divided into four types according to the various regulatory characteristics (**Table 1**), the first of which is composed of AC1, AC3, and AC8, which are activated by cationic calcium (Ca^2+^) and calmodulin (CAM). The second involves AC2, AC4, and AC7, which are activated by heterotrimeric GTP-binding protein (G-protein) βγ subunits, while they are insensitive to calcium. The next group, consisting of AC5 and AC6, is inhibited by Ca^2+^ and inhibitory G-protein (Gi), and AC9, as the only member of the fourth type, is the only isomer that is not activated by forskolin, while it responds to calcineurin (15). In addition, sAC is stimulated by bicarbonate, calcium, and ATP, while it is insensitive to forskolin and heterotrimeric G-protein (16). ACs serve as the effectors of the G-protein-coupled system. Furthermore, at least two heterotrimeric G-proteins are responsible for regulating the activity of transmembrane adenylate cyclase (tmAC), which include stimulatory G-protein (Gs) and Gi, which activate and inhibit AC, respectively (17). It is worth noting that the differential regulation and unique distribution of various AC isomers can create completely different cellular signals, making them key enzymes in the signaling pathway (18). ACs are widely distributed, the diverse subtypes of which perform various physiological functions, and each subtype has nonspecific cross-coordination or antagonism properties. The results of some studies demonstrated that the regulation of ADCY expression is closely related to animal health, while it lacks effective selectivity (19). Furthermore, the nonselective inhibition of ACs causes a series of potential adverse reactions like long-term memory (LTM) impairment (AC1 and AC8), motor dysfunction (AC3), nephrogenic diabetes insipidus (AC6), renal function decline (AC3, AC5, and AC6), and higher mortality rate during sympathetic nerve maintenance stimulation (other ACs) (19).

**Table 1:** Activators or inhibitors for AC subtypes and related diseases.

Type of AC		Coded by	Related diseases	Activator^a^	Inhibitor^b^	Insensitive^c^
**Membrane-bound AC**						
Type 1	AC1	ADCY1	Long-term memory impairment	Ca^2+^; CAM and forskolin		
	AC3	ADCY3	Motor dysfunction; renal function decline; obesity; diabetes			
	AC8	ADCY8	Long-term memory impairment; neuropsychiatric disorders			
Type 2	AC2	ADCY2	Breathing dysfunction; neuropsychiatric disorders; two-way emotional disorder	Heterotrimer G protein βγ subunits; forskolin		Calcium
	AC4	ADCY4	Breast cancer			
	AC7	ADCY7	Autoimmune diseases; depression			
Type 3	AC5	ADCY5	Renal function decline; alcohol addiction; extrapyramidal movement disorders	Forskolin	Ca^2+^,Giα	
	AC6	ADCY6	Nephrogenic diabetes insipidus; renal function decline; heart failure			
Type 4	AC9	ADCY9	Stroke in sickle cell disease; immune function disorders; different cancer	Calcineurin		Forskolin
Soluble AC						
Type 1	sAC	ADCY10	Prostate cancer; breast cancer; glaucoma; diabetes	Bicarbonate, calcium and ATP		Forskolin, heterotrimer G protein

AC/ADCY, adenylate cyclase; CAM, calcium-regulated proteins; Giα, the α subunit of the Gi protein; ATP, adenosine triphosphate.

a Activator: the ADCY subtype of this classification is stimulated under the influence of such substances, thus activating the relevant downstream molecules for a subsequent series of reactions.

b Inhibitor: the ADCY subtype of this classification is inhibited under the influence of such substances, thereby reducing the occurrence of related downstream molecules and the subsequent series of reactions.

c Insensitive: the ADCY subtypes of this classification are sensitive to such foreign substances and can stimulate the ADCY subtype to react accordingly.

cAMP-related pathways and biological function.

The cAMP directly regulates many cellular functions (20). Intracellular cAMP is produced from ATP during a reaction catalyzed by ACs, while cAMP degradation is mediated by cAMP phosphodiesterase (PDE), which hydrolyses cAMP to produce adenosine 5′-monophosphate (21). Thus, the dynamic balance of intracellular cAMP levels is maintained by a combination of AC stimulation and PDE-mediated degradation. Additionally, cAMP acts as an intracellular signal for many hormones, neurotransmitters, and other signaling molecules, the production and degradation of which are accompanied by its sensitivity to a wide range of extracellular and intracellular signals. Some heterologous G-protein-coupled receptors (GPCR) such as nicotinic acetylcholine, beta-adrenergic, or adenosine receptors activate AC, leading to a rise in cAMP production (22). Similarly, forskolin directly stimulates AC, and theophylline and caffeine promote intracellular cAMP levels by inhibiting PDE (16), and consequently affect a range of pathophysiological functions.

## 3 AC-Related Disorders

Today, AC subtypes are known to be associated with different diseases (**Table 1**), such as autoimmune ones and depression (23), as well as stroke in sickle cell disease, immune function disorders, and different cancers (AC9) (24). Furthermore, AC1 and AC4 are respectively accompanied by LTM impairment (25) and breast cancer (26), while AC3 is related to motor dysfunction, renal function decline, obesity, and diabetes (18). An association is found between AC8 with LTM impairment and neuropsychiatric disorders (27). Some researchers found a relationship between AC2 with breathing dysfunction and neuropsychiatric and bipolar disorders (28), as well as another between AC5 with renal function decline, alcohol addiction, and extrapyramidal movement disorders (29). Furthermore, AC6 is accompanied by nephrogenic diabetes insipidus, renal function decline, and heart failure (30). Finally, sAC is associated with prostate and breast cancer, glaucoma, and diabetes (31). Thus, AC subtypes are implied as important players in various diseases, suggesting that relevant diseases can be controlled by the specific targeting and regulation of AC isoforms.

## 4 cAMP-RELATED PATHWAYS AND BILOGICAL FUNCTION

**Figure 2:**
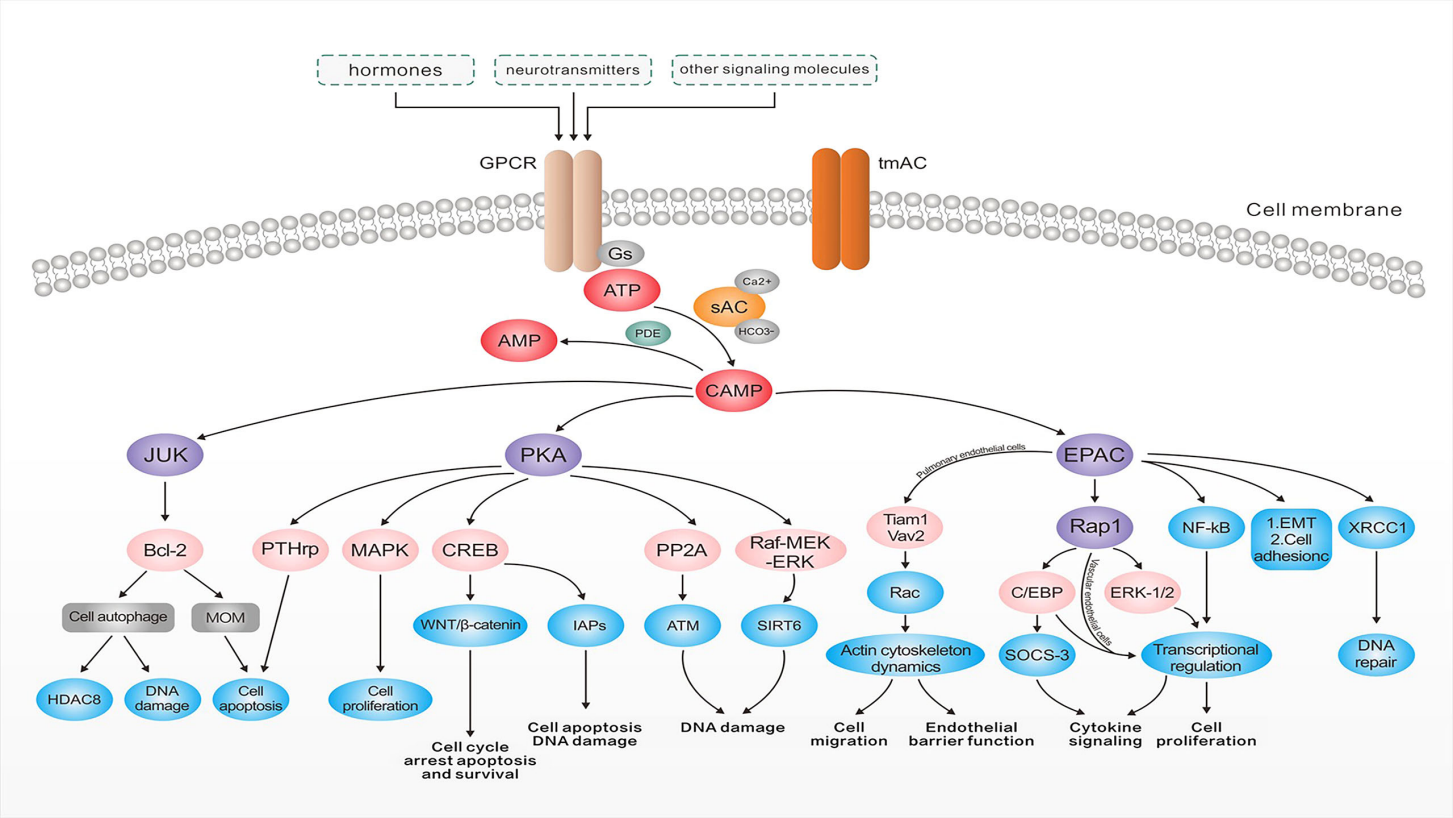
The roles of adenylate cyclases in tumorigenesis. After binding to G protein-coupled receptors, hormones, neurotransmitters, and a number of other signaling molecules, cyclic adenosine 3′,5′-monophosphate (cAMP) is generated from ATP by the action of adenylate cyclase and degraded to the 5′-cAMP by the action of cyclic nucleotide phosphodiesterase (PDE). cAMP can increase DNA damage *via* a range of pathways and plays an important role in endoskeletal remodeling, cell proliferation, adhesion, and EMT. It also regulates the permeability of the mitochondrial outer membrane, affecting cell survival, activates the WNT/β-catenin pathway, and regulates the processes of cell cycle arrest, apoptosis, survival, and DNA damage. cAMP signaling pathways can also protect cancer cells from apoptosis after exposure to radiation or chemotherapeutic agents through related pathways. Abbreviations: EGFR, epidermal growth factor receptor; tmAC, transmembrane adenylate cyclase; Gs, stimulatory G-protein; sAC, soluble adenylate cyclase; ATP, adenosine triphosphate; PDE, phosphodiesterase; cAMP, cyclic adenosine phosphate; PKA, protein kinase A; JNK, c-Jun N-terminal kinase; Bcl-2, B-cell lymphoma-2; MOM, mitochondrial outer membrane; HDAC8, histone deacetylase 8; MAPK, mitogen-activated protein kinase; CREB, cAMP response element-binding proteins; IAPs, inhibitor of apoptosis proteins; PP2A, protein phosphatase 2A; ATM, the ataxia–telangiectasia–mutated protein; Raf-MEK-ERK, Raf/mitogen-activated extracellular signal-regulated kinase/extracellular signal-regulated kinase; EPAC, cAMP-EGF-I/II; ERK, extracellular signal-regulated kinase; XRCC1, X-ray repair complementing defective repair in Chinese hamster cell 1; EMT, epithelial-mesenchymal transition; SOCS-3, suppressor of cytokine signaling 3; C/EBP, CCAAT/enhancer-binding protein.

The cAMP, as the first identified second messenger, regulates cellular transport by binding to PKAs, cAMP-activated guanine exchange factors (EPACs), and cyclic nucleotide-gated channels (CNGs) (32) (**Figure 2**). These cAMP sensors lead to the regulation of various physiological processes such as DNA degradation, cell proliferation, and apoptosis either alone or in cooperation with PKAs (33). PKAs and EPACs are the major targets of the cAMP signaling pathway (34). In addition, activated PKA-phosphatases regulate related metabolic and transcription factors like the activation of downstream cAMP response element-binding proteins (CREB) and consequently activate the WNT/β-catenin pathway, which is accompanied by cell cycle arrest, apoptosis, and survival (34). CREB regulates the inhibitory activity of apoptosis proteins (IAPs) to prevent apoptosis triggered by various stimuli and plays an important role in inhibiting cell death and regulating DNA damage in the cell cycle (35). Furthermore, the cAMP signaling pathway regulates sirtuin 6 expression through modulating the PKA-dependent Raf/mitogen-activated extracellular signal-regulated kinase/extracellular signal-regulated kinase (Raf-MEK-ERK) pathway as well as ubiquitin-protease-dependent degradation, causing apoptosis regulation (36). Similarly, the regulation of protein phosphatase 2A (PP2A) plays a crucial role in regulating apoptosis (37). PKA activation can protect cancer cells from apoptosis after exposure to radiation or chemotherapeutic agents by increasing parathyroid hormone-related protein expression (38). The higher PKA expression enhances the activity of the mitogen-activated protein kinase (MAPK) signaling pathway (39), which regulates cell proliferation. The results of a recent study demonstrated the important role of X-ray repair in complementing defective repair in repairing DNA in Chinese hamster cell 1 (XRCC1) and the ability of cAMP-mediated EPACs to repair DNA damage by regulating XRCC1 (40). Furthermore, EPACs regulate cell proliferation, differentiation, apoptosis, and inflammation as well as play a crucial role in endoskeletal remodeling, cell proliferation, adhesion, migration, and epithelial-mesenchymal transition by regulating downstream signaling molecules in the vascular cAMP signaling pathway (40). They can even act by preventing c-Jun N-terminal kinase (JNK) activation by inhibiting B-cell lymphoma-2 (Bcl-2) phosphorylation, downregulating cellular autophagy and phosphorylation, and subsequently leading to less ubiquitination and more histone deacetylase 8 (HDAC8) expression (41). Additionally, Bcl-2 is a well-known member of the antiapoptotic protein family, which serves by inhibiting the essential proapoptotic proteins that share multiple homology domains with Bcl-2 (42). It regulates mitochondrial outer membrane (MOM) permeability, which has a key role in apoptotic signaling and influences cell survival (43). Therefore, ACs regulate cAMP production, which results in modulating downstream signaling molecules to control various cellular physiological activities.

## 5 Mutations in ADCY Family

### 5.1 Disease-Causing Effects of the Mutations in the ADCY Family

To date, at least 60 *ADCY* gene mutations have been reported in many genotype–phenotype databases, such as the Online Mendelian Inheritance in Man (OMIM) and Human Gene Mutation Database (HGMD), as well as the Database of Genotype and Phenotype (dbGAP). Mutations in ADCY1, ADCY2, ADCY3, ADCY4, ADCY6, and ADCY7 isoforms exhibit the most pathological effects and clinical significance. The disease-causing mutations are predominantly nonsense variants, partial and full deletions, and nonsense, insertion, and splice-site mutations. Nonsynonymous single nucleotide polymorphisms (nsSNPs, substitutions, and deletions) are considered the most common variants of *ADCY1* and *ADCY6*. Furthermore, exons 22 (22,896 bp of variants/length) and 23 (148,977 bp of variants/length) are the most affected exons. Some *ADCY* mutations were initially identified incorrectly due to the similarity between their symptoms and the other cancer-driver mutations. **Table 2** lists the most frequent disease-causing SNP mutation spectrums in *ADCY1* and *ADCY6*. As shown, the nonsynonymous mutations of G>A are more widely observed and change highly conserved amino acids in ADCY1 and ADCY6. The deleterious and pathogenic aspects of mutations were predicted by using an *in silico* prediction pipeline consisting of polymorphism phenotyping v2 (PolyPhen-2), mutation taster, I-Mutant, sorting intolerant from tolerant (SIFT), and ExAC programs. Furthermore, several online programs were applied to predict the pathogenicity of the selected variants of ADCY1 and ADCY6. Based on the predictions, *ADCY6* variants are disease-causing and damaging mutations, especially p.E1003K, p.R1116C, and p.Y992C, which were verified by more than two bioinformatics programs (**Table 2**). Three variants have a deleterious effect when using the SIFT and PolyPhen2 tools. Generally, the nsSNPs c. 3007G>A (p.E1003K), c. 3346C>T (p.R1116C), and c. 2975A>G (p.Y992C) are predicted to be pathogenic mutations with the potential as cancer-driver mutations.

**Table 2:** Predicted protein structure and disease-causing effects of mutations in AC family.

Gene	Exon	Variation				Polyphen-2 [sensitivity–specificity]^b^	Mutation taster^c^	I-Mutant3.0 (kcal/mol)^d^	SIFT^e^	EXAC^f^	Overall evaluated pathogenicity
		Nucleotide^a^	Protein^a^	Type	Status						
ADCY1	22	c.2818 G>A	p.Ala940Thr	Missense	Homo	B (0.005) [0.97–0.740]	B (1.00)	DS (−0.45)	T (0.05)	Novel	Benign
	22	c. 3090G>A	p.Val984Met	Synonymous	Homo	PD (1.000) [0.00–1.00]	B (1.00)	DS (−1.37)	NT (0.05)	Novel	Benign
	22	c. 3184G>A	p.Gly1062Ser	Missense	Homo	PD (1.000) [0.00–1.00]	B (1.00)	DS (−1.37)	T (1.00)	Novel	Benign
ADCY6	23	c. 1640T>C	p.Ile547Thr	Missense	Hetro	PD (1.000) [0.00–1.00]	B (0.60)	DS (−2.22)	NT (0.05)	Novel	Benign
	23	c. 2029C>A	p.Leu677Met	Missense	Homo	PD (0.716) [0.86–0.92]	B (0.60)	DS −1.08)	T (0.05)	Novel	Benign
	23	c. 3007G>A	p.Glu1003Lys	Missense	Homo	PD (0.989) [0.72–0.97]	De (0.60)	DS (−0.63)	NT (1.00)	Pathogenic	Pathogenic
	23	c. 3346C>T	p.Arg1116Cys	Missense	Homo	PD (1.000) [0.00–1.00]	De (0.00)	DS (−1.21)	NT (1.00)	Pathogenic	Pathogenic
	23	c. 2975A>G	p.Tyr992Cys	Missense	Hetro	PD (1.000) [0.00–1.00]	De (0.60)	DS (−0.89)	NT (0.05)	Pathogenic	Likely pathogenic

c, variation at cDNA level; G, guvanin; G, guanine; A, adenine; T, thymine C, cytosine; p, variation at protein level; Ala, alanine; Thr, tyrosine; Val, valine; Met, methionine; Gly, glycine; Ser, serine; Ile, isoleucine; Leu, leucine; Glu, glutamic acid; Lys, lysine; Arg, arginine; Cys, cysteine; Tyr, tyrosine; Homo, homozygote; Hetro; heterozygous; B, benign; PD, probably damaging; DC, disease-causing; De, deleterious; DS, decrease stability; NT, not tolerated; T, tolerated.

a All nucleotide and amino acids are abbreviated according to the International Union of Pure and Applied Chemistry (IUPAC).

b Polymorphism phenotyping v2 (Polyphen-2) is used to predict the possible impact of amino acid substitutions on the stability and function of proteins using structural and comparative evolutionary considerations.

c Mutation taster is applied to evaluate the disease-causing potential of sequence alterations.

d I-Mutant3.0 support vector machine (SVM)-based tools were used for the automatic prediction of protein stability changes upon single-point mutations

e Sorting intolerant from tolerant (SIFT) program is used to predict whether an amino acid substitution affects protein function so that users can prioritize substitutions for further study.

f ExAC databases were used to identify individuals expected to exhibit a childhood disorder based on concordance with disease inheritance modes: heterozygous (for dominant), homozygous (for recessive), or hemizygous (for X-linked recessive conditions).

### 5.2 Functional Effects of a Pathogenic Mutation in the ADCY Family

**Figure 3:**
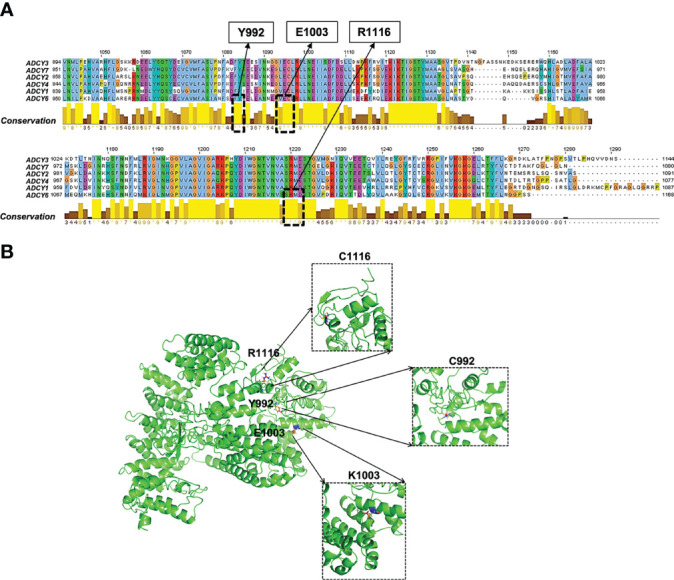
Functional effects of pathogenic *ADCY6* SNPs. **(A)** Multiple sequence alignment of the ADCY protein family. **(B)** Surface and crystallographic imaging of the ADCY6 p.E1003K and p.R1116C SNPs located in the active site of the ADCY6 enzyme.

The analysis of the conserved domains of wild-type (WT) and mutant ADCY6 proteins is shown in **Figure 3**. In addition, the mutation sites were aligned to the ADCY6 protein sequence to explore the function-associated nsSNPs (**Figure 3A**). Most of the disease-driver mutations generate defective helicase proteins due to the changes in conserved residues (i.e., Y991, E1003, and R111), as well as the degradation of the improperly folded protein or truncated mRNA. Structural–functional analysis revealed that all three disease-driver nsSNPs (p.E1003K, p.R1116C, and p.Y992C) are located in the topological domain of the ADCY6 protein (914–1,168 amino acid residues in the cytoplasmic domain). Based on the sequence conservation alignment, the nsSNP Y991, E1003, and R1116 are highly conserved in all organisms (**Figure 3A**). As shown in **Figure 3B**, the predicted crystal structures of the WT (left panel), and E1003K, R1116C, and Y992C mutant (right panel) ADCY6 proteins exhibit the mutation of a glutamic acid residue to lysine, arginine to cysteine, and tyrosine to cysteine at conserved positions 1003, 1116, and 992, respectively. Structurally, the active site is situated at the interface of the two topological domains, locating the three residues in the ADCY6 enzyme active site. The comparison between the protein structure homology-modeling of the WT and ADCY6 mutant (p.E1003K) demonstrated a change from a smaller Glu to a larger Lys at position 1003, leading to charge variation (positive charge). However, the other mutations result in introducing cysteine residues, which form disulfide bonds in the protein structure. Furthermore, the WT and mutant proteins were significantly different in terms of hydrophobicity and hydrophilicity. Therefore, the mutation may cause the loss of hydrophobic interactions and hydrogen bonds with other molecules, which alters the enzyme activity. In general, ADCY6 catalyzes the formation of the signaling molecule cAMP downstream of G-protein-coupled receptors, which act in the signaling cascade downstream of beta-adrenergic receptors in the heart and vascular smooth muscle cells. Given the crystal structure, the p.E1003 and p.R1116C substitutions are suggested to trigger a change in substrate conformation slightly, with the potential to affect the enzyme active center. In the present review, it is hypothesized that the ADCY6 (p.E1003 and p.R1116C) mutant loses its activity and attenuates the hydroxylation of substrates such as those in the cAMP-PKA-PP2A (37) and WNT/β-catenin pathways.

## 6 Crosstalk Between ADCYs and Cancer

**Figure 4:**
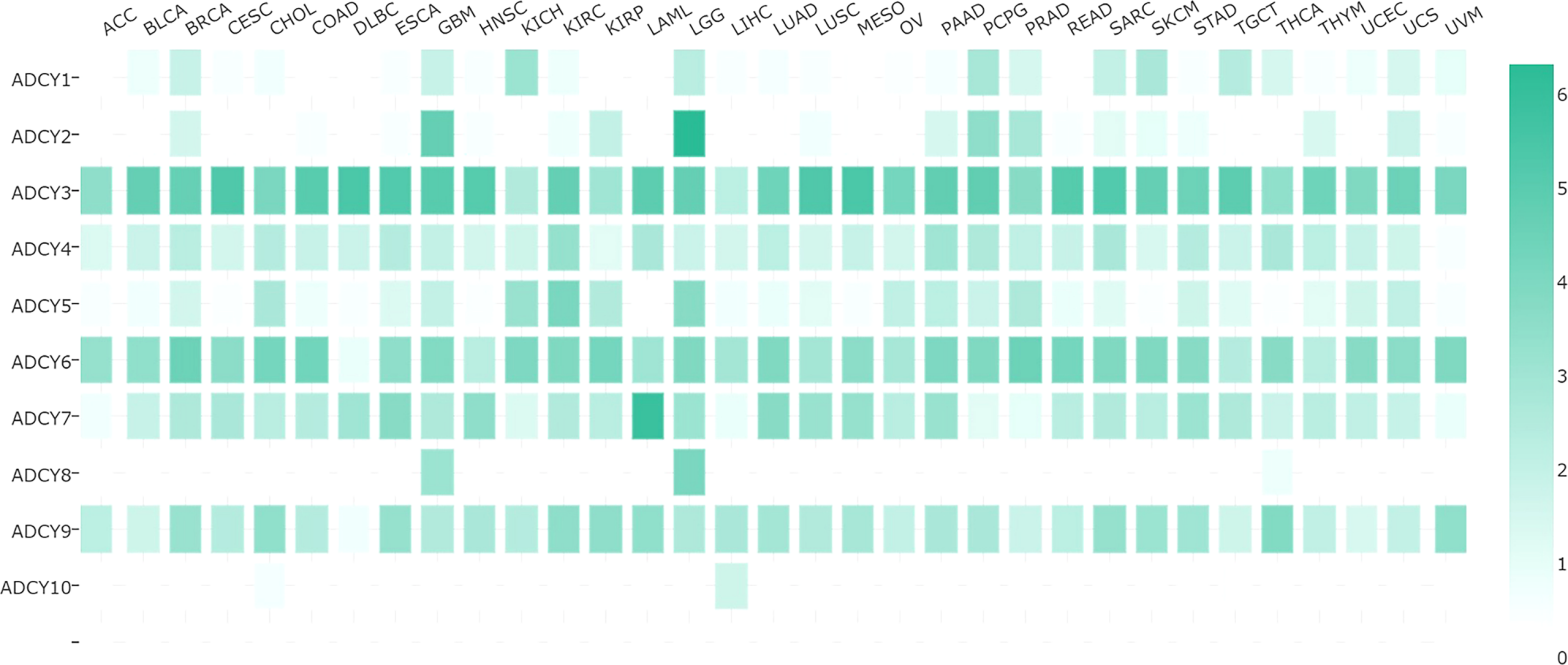
Comparison of the expression of various *ACDY* isoforms in different cancers. The adenylate cyclase gene is expressed differently in each cancer, with darker color indicating higher expression and vice versa. ACC, adrenocortical carcinoma; BLCA, bladder urothelial carcinoma; BRCA, breast invasive carcinoma; CESC, cervical squamous cell carcinoma and endocervical adenocarcinoma; CHOL, cholangiocarcinoma; COAD, colon adenocarcinoma; DLBC, lymphoid neoplasm diffuse large B-cell lymphoma; ESCA, esophageal carcinoma; GBM, glioblastoma multiforme; HNSC, head and neck squamous cell carcinoma; KICH, kidney chromophobe; KIRC, kidney renal clear cell carcinoma; KIRP, kidney renal papillary cell carcinoma; LAML, acute myeloid leukemia; LGG, brain lower-grade glioma; LIHC, liver hepatocellular carcinoma; LUAD, lung adenocarcinoma; LUSC, lung squamous cell carcinoma; MESO, mesothelioma; OV, ovarian serous cystadenocarcinoma; PAAD, pancreatic adenocarcinoma; PCPG, pheochromocytoma and paraganglioma; PRAD, prostate adenocarcinoma; READ, rectum adenocarcinoma; SARC, sarcoma; SKCM, skin cutaneous melanoma; STAD, stomach adenocarcinoma; TGCT, testicular germ cell tumors; THCA, thyroid carcinoma; THYM, thymoma; UCEC, uterine corpus endometrial carcinoma; UCS, uterine carcinosarcoma; UVM, uveal melanoma.

The development, progression, metastasis, and drug resistance of most common cancers are mediated by the cAMP signal downstream of β-adrenergic receptors (β-ARs), which are coupled to stimulatory G-protein (Gs) (44). After hormones, neurotransmitters, and some other signaling molecules bind to G-protein-coupled receptors (**Figure 4**). Additionally, ATP is generated from cAMP through the action of AC and then involved in a range of pathophysiological activities. In contrast, 3′,5′-cAMP is degraded to 5′-cAMP by the action of PDE. The cAMP plays an important role in endoskeletal remodeling, cell proliferation, adhesion, and epithelial–mesenchymal transition (EMT) by preventing JNK activation, leading to the inhibition of Bcl-2 phosphorylation and, consequently, downregulation of cellular autophagy and phosphorylation. As a result, ubiquitination decreases, HDAC8 expression increases, and DNA damage increases (41). Furthermore, Bcl-2 regulates MOM permeability, which has a significant role in apoptotic signaling and affects cell survival (45). Activated PKA-phosphatases, which regulate related metabolic and transcription factors like CREB (34), are associated with cell cycle arrest, apoptosis, and survival through activating the WNT/β-catenin pathway. They control apoptosis and DNA damage by regulating the enhancer sequences of IAPs (35). Furthermore, the cAMP signaling pathway controls apoptosis and DNA damage by regulating the PKA-dependent Raf-MEK-ERK pathway as well as protecting cancer cells from apoptosis following exposure to radiation or chemotherapeutic agents by elevating the expression of parathyroid hormone-related proteins (38). A rise in PKA improves the activity of the MAPK signaling pathway, which regulates cell proliferation (39). In addition, EPACs regulate cell proliferation, migration, adhesion, EMT, and endoskeletal remodeling by regulating downstream signaling molecules. The cAMP-mediated EPACs can repair DNA damage by regulating XRCC1 (40).

In this regard, AC activates the formation of intracellular cAMP-activated PKA and transcription factor CREB, which induces the overexpression of growth factors such as epidermal growth factor (EGF), VEGF, arachidonic acid (AA), and proinflammatory cytokines, and consequently stimulates the growth, development, metastasis, and drug resistance of many cancers (44). Furthermore, the Gi-coupled receptor can inhibit this signaling cascade by blocking the activation of the ACs catalyzing cAMP formation, and acting as a physiological inhibitor of the cascade (44). Thus, AC overexpression or silencing may be a key event in processes like tumorigenesis, cell proliferation, migration, and invasion.

### 6.1 ADCY Isoforms in Tumorigenesis

The cAMP-mediated activation of PKA in many cancer cells leads to cell cycle arrest and growth inhibition *via* the apoptotic pathway (46). The blocking of ERK (47), inhibition of antiapoptotic proteins Bcl2 and Bcl-xl (48), upregulation of tumor suppressor gene p53, and suppression of oncogenes c-myc and erb-2 (49) can be addressed as other ways in which cAMP is linked to growth inhibition. Furthermore, the regulation of the angiogenic pathway through preventing VEGF, transforming growth factor-β (TGF-β), and EGFR is associated with higher cAMP levels (50). However, a lower level of ADCY and cAMP is found in many cancers, which promotes cancer formation and cancer cell proliferation, decreases apoptosis, and even increases neovascularization, cancer cell migration, and invasion.

### 6.2 ADCY Family in Cancer Recurrence and Prognosis

**Figure 5:**
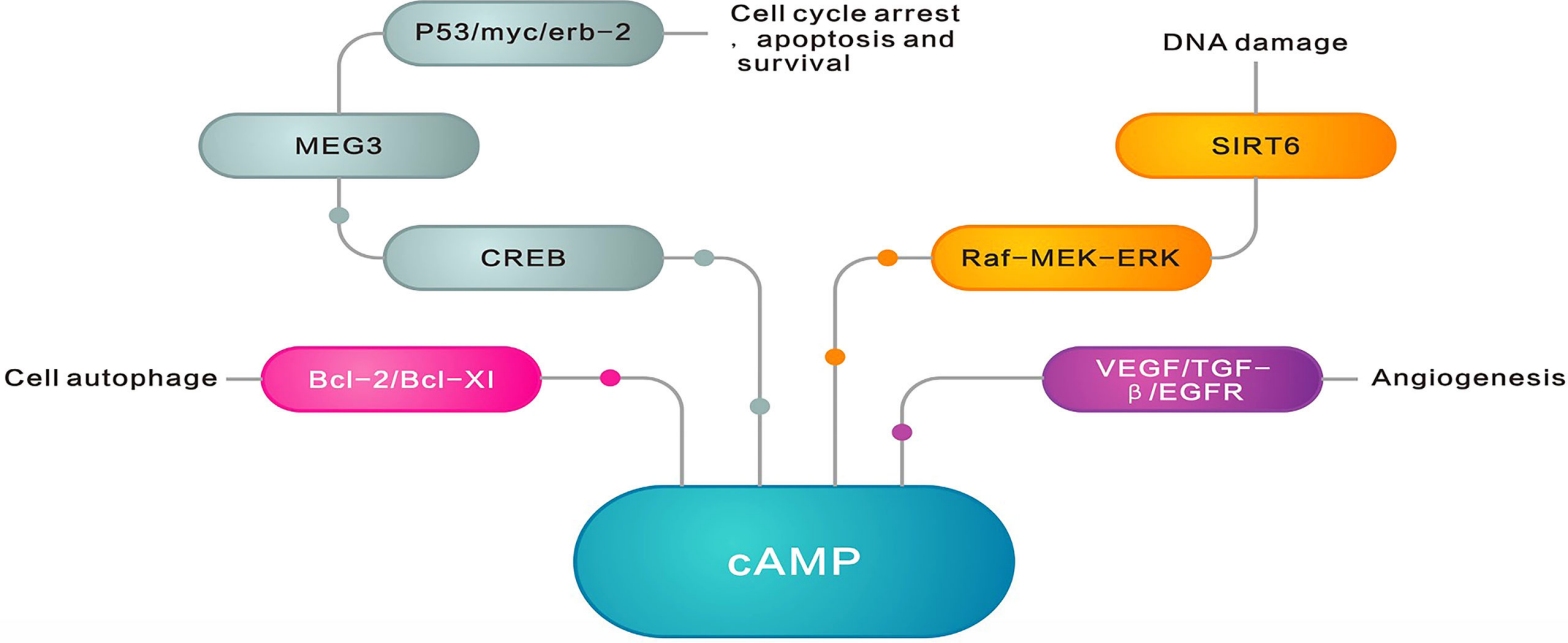
ADCY family roles in cancer recurrence and prognosis. cAMP inhibits cancer formation by blocking extracellular signal-regulated kinase (ERK), inhibiting the antiapoptotic proteins Bcl2 and Bcl-XI, upregulating the cancer suppressor gene p53, inhibiting the oncogenes c-myc and erbB-2, and inhibiting the regulation of the angiogenic pathway by VEGF, TGF-β, and EGFR. However, many cancers exhibit lower AC levels and lower cAMP levels, promoting cancer formation as well as cancer cell proliferation, reducing apoptosis, and even promoting neovascularization, cancer cell migration, and invasion.

As already mentioned, the ADCY family is closely related to cancer recurrence and prognosis (**Figure 5**). Cancer recurrence is a common phenomenon involving numerous complex mechanisms, in which the ADCY family plays a crucial role. The results of the previous studies indicated less AC7 expression among relapsed acute promyelocytic leukemia (APL) patients than the newly diagnosed APL ones. Additionally, miR-192, which directly targets AC7 expression, is relatively high in the relapsed APL individuals, which suggests the important role of miR-192-mediated AC7 in APL cell differentiation, as well as implicating AC7 and miR-192 as novel biomarkers and therapeutic targets for these patients (51). Individuals with primary or relapsed APL have a relatively lower level of AC9 expression compared to those with complete remission and nonleukemic patients (52). Furthermore, the expression is strongly accompanied by leukemogenesis in APL, proposing the potential of AC9 as a biomarker in clinical diagnosis and leukemia relapse treatment (52). The less expression of phosphorylated CREB protein produced through cAMP regulation is associated with the aggressive and metastatic recurrence of melanoma (53). Moreover, the ADCY family, as the key genes in important signaling pathways, influences patient prognosis through a variety of mechanisms. Based on the results of a cohort study, the level of ADCY9 is greater in colon cancer tissues than in the adjacent tissues (54). The low ADCY9 level in cancer tissues is attributed to longer disease-free survival (DFS), while high ADCY9 expression and distant metastasis indicate a poor prognosis after treatment (54).

### 6.3 ADCY Family in Chemo- and Radioresistance

Many treatment options are available for cancer, which include surgery, chemotherapy, radiotherapy, molecular targeted therapy, and immunotherapy. Specific cancer cell death induction and maximum remission are the ultimate goals of anticancer therapy. Currently, these goals still rely mainly on drug- or radiation-induced DNA-triggered death and antiproliferative signals. AC is activated by cAMP, which in turn activates PKA and CREB, which have roles in gene regulation, cell migration and proliferation, apoptosis, and mitochondrial homeostasis (55). In addition, forskolin, as an AC agonist, exerts several relevant anticancer effects such as inducing EMT, enhancing sensitivity to conventional antitumor drugs, and inhibiting the proliferation, motility, and migration of many types of cancer cells (50, 56).

In lung cancer, ADCY1 can modulate anticancer drugs and regulate apoptosis by regulating the expression of Bcl-2 family proteins, IAPs, and XRCC1 in human lung cancer cells (33). The expression of apoptosis inhibitors and inactivation of apoptosis promoters have been reported in human cancers. The results of the preclinical models suggested that functional defects in apoptosis signaling may translate into drug-resistant cells in cancer. The MOM permeability is known as a key step in apoptotic signaling, which is regulated by the Bcl-2 family of proteins, and targeting BCL in nonsmall cell lung cancer treatment leads to an initial improvement in chemoresistance (57). Further, ADCY1 catalyzes the elevation of cAMP and consequently inhibits the EPAC-dependent pathway degradation of XRCC1-induced DNA damage, DNA repair, and apoptosis in lung cancer cells (58). Ataxia-telangiectasia mutated (ATM) is a master regulator of the cellular response to the ionizing radiation-induced DNA damage, which can regulate multiple DNA damage responses like cell cycle, DNA repair, and apoptosis through activating downstream signaling pathways (59, 60). Gαs simulates ATM activation through the Gαs-cAMP-PKA-PP2A pathway, which results in declining the ATM protein-dependent nuclear factor kappa-B (NF-κB) activation, and increasing radiation-induced apoptosis in human and mouse lung cancer cells (37). Furthermore, the downregulation of cAMP-regulated Meg3 (an RNA gene) promotes cisplatin resistance in lung cancer cells by activating the WNT/β-catenin signaling pathway (61).

Breast cancer resistance to chemotherapeutic agent doxorubicin is generally accompanied by RAS/RAF/ERK activation (62). Additionally, AC activation can sensitize triple-negative breast cancer (TNBC) cells to doxorubicin *via* the cAMP/PKA-mediated mechanism of ERK inhibition, which greatly elevates doxorubicin-induced cell death (62). Forskolin can improve the sensitivity of MDA-MB-231 and MDA-MB-468 TNBC cells to 5-fluorouracil and taxol l (63).

Further, the cAMP signaling system inhibits DNA damage-induced apoptosis in leukemic cells by promoting p53 acetylation and turnover. The results of a study reflected an association between the upregulation of multidrug resistance gene (MDR1) expression through activating CREB by PKA with multidrug resistance in leukemia cells (64). EPAC1 and EPAC2 exhibit different expression patterns in mature and developing tissues. EPAC1 mRNA is widespread (65), while EPAC2 is mainly expressed in brain and endocrine tissues (66). Recently, some researchers have proposed the involvement of EPAC1 in regulating a variety of cancer cellular responses like cancer cell adhesion (67), proliferation (68), invasion (69), and migration (69, 70). Regarding ovarian cancer cells, roflumilast induces apoptosis and prevents tumor progression by activating the cAMP/PKA/CREB pathway and upregulating mitochondrial ferritin (FtMt) levels in the two types of cells (OVCAR3 and SKOV3) (55). Furthermore, ADCY4 is suggested to be accompanied by cetuximab insensitivity in colorectal cancer (71).

**Table 3** presents an available spectrum of the multifunctional genes upregulated by ADCY in various radioresistant tumor cells. ADCY1 can modulate anticancer drugs and regulate cell apoptosis *via* the Bcl-2-MOM pathway in lung cancer (57). It can modulate cell apoptosis (35) and EPAC-XRCC1 through IAPs, both of which induce DNA damage (by increasing cell insensitivity to γ-ray), DNA repair, and apoptosis (40). In addition, it can enhance radiation-induced cell apoptosis *via* the cAMP-PKA-PP2A-ATM-NF-κB pathway (37) and cause more resistance to drugs such as cisplatin through the cAMP-Meg3-WNT/β-catenin pathway (61). All of the pathways in lung cancer involve changes in the sensitivity and effectiveness of chemotherapeutic drugs and radiotherapy. In breast cancer, it enhances the sensitivity of cancer cells to doxorubicin by improving the PKA-mediated activation of ERK to induce cell apoptosis and consequently promote the efficacy of the associated chemotherapy (63). Furthermore, it leads to the modulation of DNA damage-induced apoptosis *via* the cAMP-p53 pathway and cAMP/PKA/CREB1-MDR1 signaling axis in leukemia and is accompanied by multidrug resistance (64). In the case of ovarian cancer, it induces cell apoptosis and increases sensitivity to cisplatin, even reverses cisplatin resistance, through the cAMP/PKA/CREB-FtMt signaling axis (55). In general, the ADCY family results in improving the efficacy of cancer therapy because of modulating the downstream pathways which alter the sensitivity of cancer cells to chemotherapeutic agents and radiotherapy.

**Table 3:** Role of adenylate cyclase-related pathways in cancer drug or radiotherapy sensitivity.

Types of cancer	Signaling axis of action	Cellular function	Drug/radial sensitivity	Phenotype function	References
Lung cancer	bcl-2-MOM	Modulate anticancer drugs and regulate apoptosis			[Willis et al. (45) and Mansilla Pareja et al. (40)]
	IAPs	Cell apoptosis			[Mansilla Pareja et al. (40)
	Epac-XRCC1	Induce DNA damage, DNA repair, and apoptosis	γ-ray		Mansilla Pareja et al. (40)]
	cAMP/PKA/PP2A-ATM-NF-κB	Enhance radiation-induced cell apoptosis			[Mansilla Pareja et al. (40)]
	cAMP-Meg3-WNT/β-catenin	Enhance drug resistance	Cisplatin		[Xia et al. (61)]
Breast cancer	cAMP/PKA/ERK	Induce cell apoptosis	Doxorubicin		[Abrams et al. (72)]
Leukemia	cAMP-p53	DNA damage-induced apoptosis			[Naderi et al. (73)] and [Kloster et al. (74)]
	cAMP/PKA/CREB1-MDR1			Multidrug resistance	(64)
Ovarian cancer	cAMP/PKA/CREB-FtMt	Induce cell apoptosis	Cisplatin	Reverse cisplatin resistance	[Gong et al. (55)]

Bcl-2, B-cell lymphoma-2; MOM, mitochondrial outer membrane; IAPs, inhibitor of apoptosis proteins; EPAC, cAMP-EGF-I/II; XRCC1, X-ray repair complementing defective repair in Chinese hamster cells 1; PP2A, protein phosphatase 2A; ATM, the ataxia–telangiectasia–mutated protein; NF-κB, nuclear factor kappa-B; PKA, protein kinase A; ERK, extracellular signal-regulated kinase; CREB, cAMP response element-binding proteins; MDR1, multidrug resistance gene.

### 6.4 Role of ADCY Isoforms in Various Cancers

The expression of ADCY subtypes varies in various cancers so that some are highly expressed in tumors and participate in tumor formation and development as oncogenes, while the others may have low, or even no expression. Analyses have revealed that this variation may lead to antitumor effects and may even be related to tumor prognosis. The expression of each subtype of the ADCY family in different cancers is demonstrated in **Figure 4**. As displayed, the most significant variation in expression is observed in ADCY4 and ADCY9. ADCY4 mRNA expression is significantly downregulated in breast cancer compared to the normal tissues, while the level more than triples in invasive breast cancer. Furthermore, ADCY9 expression diminishes in colorectal and lung cancers in comparison with the normal tissues (26). The epigenetic changes in the ADCY family, as a gene family regulating the extensive and global physiological activities of organisms, play a certain role in the biological behavior of tumors and other pathological processes. The results of recent gene sequencing-based studies have introduced a new role for *ADCY1* mutations in influencing drug effectiveness in a variety of cancers like lung, esophageal, and colorectal ones (33). However, *ADCY4* is significantly silenced and highly methylated in many cancers. *ADCY5* has been less well studied, and the results of research knocking out the *AC5* gene in mice indicated a significant reduction in angiogenesis and a rise in cancer cell apoptosis, both of which prevent cancer growth (75). ADCY6 expression is negatively correlated to the signaling pathways associated with immune processes, as well as the activation of immune checkpoint receptors and ligands (76). It is a potentially key gene in regulating immune cell infiltration in luminal-like carcinomas (76). **Table 3** lists the cancers in which the AC subtype has been investigated so far.

#### 6.4.1 Digest System Cancer

In gastric cancer cell lines and tissues, ADCY3 overexpression promotes tumorigenesis by upregulating the expression of matrix metalloproteinase-2 (MMP2) and metalloproteinase-9 (MMP9) *via* the cAMP/PKA/CREB pathway, which increases cell migration, invasion, proliferation, and colony formation (77, 78). *ADCY3* serves as an oncogene in gastric cancer formation, as an oncogene, with high ADCY3 expression regulated by DNA methylation accompanied by low survival (78). Additionally, the *ADCY3* gene is more expressed in primary pancreatic cancer and precancer tissues than the normal pancreatic tissues, while the expression of the *ADCY2* gene is downregulated in primary tumors and adjacent nontumor tissues (79). Unlike gastric cancer, a greater cAMP level in pancreatic cancer inhibits cell migration and invasion (80, 81), although it does not affect cell proliferation (81). In fact, AC3 may enhance cAMP levels in response to forskolin stimulation, which causes PKA to phosphorylate CREB, a primary binding protein for the cAMP response, and consequently inhibits cell migration and invasion. Its inhibitory effect is accompanied by the rapid formation of AC3/adenylate cyclase-associated protein 1 the (CAP1)/G-actin complex, which prevents filopodia generation and cell motility (79).

Certain subtypes of the ADCY family (ADCY2, 3, 8, and 9) are mutated in hepatocellular carcinoma, which may be key events in the formation of the carcinoma (82). The results of the genomic analysis of a patient with lung metastasis from colorectal cancer suggested ADCY2 and ADCY9 as potential metastasis prognostic biomarkers (83).

#### 6.4.2 Breast Cancer

Based on the results in **Figure 4**, ADCY4 expression significantly decreased in breast cancer. The results of the previous studies have shown that Giα is highly expressed in breast cancer cells, especially in metastatic ones. A potential relationship is found between ADCY4 and Giα (7, 84). Furthermore, the proximal targets of cAMP are proteins with diverse functions, which regulate the transcription rates of many genes, such as those playing a role in EMT and inhibiting cell growth and migration, as well as anticancer drug transcription factors with biological roles in anticancer drug sensitivity (7, 84). Unlike gastric cancer, ADCY4 may impose a tumor inhibitory effect in breast cancer, although this effect is terminated by the gene silencing caused by DNA methylation (26). Thus, ADCY4 is a potential biomarker and therapeutic target to predict the prognosis of human breast cancer.

Furthermore, ADCY6 may be implicated in breast cancer progression and affect breast cancer prognosis through calcium-regulated immune and molecular signaling pathways (85). An association is observed between downregulated *ADCY6* gene expression and hypomethylation with a better prognosis in breast cancer patients (76).

Some researchers referred to the need for AC8 in breast cancer cell migration as well as the important role of the cAMP-PKA pathway in migrating MCF7 and MDA-MB-231 TNBC cells (86). In MDA-MB-231 cells, AC8 is required for focal adhesion kinase (FAK) phosphorylation, which may explain the role of AC8 in cell migration (87). AC8 and Orai1 interact to trigger the phosphorylation of Orai1 in MDA-MB-231 TNBCs, which results in inactivating Orai1 and stimulating breast cancer migration (86).

#### 6.4.3 Leukemia

Previous studies have revealed the effectiveness of ADCY7 on intracellular cAMP levels and all-trans retinoic acid (ATRA)-induced cell differentiation in APL. The inhibition of ADCY7 elevates apoptosis, reduces cell growth, and declines c-myc expression (88). ADCY7 knockdown significantly inhibits inhibitor-mediated increases in CD11b expression and proliferation in NB4, an APL cell line (51). In addition, microRNA-192 (miRNA) directly targets AC7 expression, the knockdown of which promotes ATRA-induced APL cell differentiation by regulating AC7 expression (51).

Regarding acute myeloid leukemia (AML), CD300A (a type I transmembrane protein) is expressed in the myeloid cells (89). Furthermore, lymphoid lineages cause more proliferation and migration of U937 cells by improving the expression of platelet endothelial cell adhesion molecule (PECAM1) and ADCY7 and by activating the AKT/mTOR (autophagy classical pathway) signaling pathway (89).

#### 6.4.4 Lung Cancer

ADCY1 is overexpressed in nonsmall cell lung cancer (90) and is only accompanied by patient prognosis. Furthermore, ADCY1-mediated cAMP can regulate multidrug resistance in lung cancer and other malignancies by regulating the specific long noncoding RNAs (lncRNAs) involved in different signaling pathways (33).

#### 6.4.5 Melanoma

The low expression of miR-23a-3p (a miRNA) in a melanoma cell line is significantly related to poor prognosis, which significantly diminishes overall survival (OS) and DFS. MiR-23a-3p inhibits cAMP and MAPK signaling pathways because of its targeting of ADCY1 to prevent melanoma cell proliferation, migration, invasion, and tumorigenesis (9). Additionally, anal melanoma (MIS) is the most common malignant melanoma in the East Asian population. The sAC is mainly expressed in nuclei and nucleosomes, such as the deeply stained nuclei of melanoma cells, especially MIS, as well as the protruding nuclei in the vesicular nucleus (91). It may be associated with melanoma invasion, and cytological and nuclear changes in limbs end in melanoma progression (91). However, sAC can be implicated in melanocyte proliferation, apoptosis, and melanin synthesis through its catalytic product cAMP (91).

#### 6.4.6 Laryngeal Cancer

In laryngeal cancer, ADCY6 is a biomarker with significantly different expressions in cancer and noncancer samples. Some researchers reported the involvement of ADCY6 in the cellular processes related to the cancer phenotype like cell cycle, apoptosis, DNA repair, protease inhibition, proteolysis, and transcriptional regulation (92).

#### 6.4.7 Glioblastoma

*ADCY8* polymorphisms are accompanied by the risk of glioma (brain tumor) in patients with neurofibromatosis type 1 (NF1), which is sex-specific (93). Based on the single nucleotide polymorphism (SNP) array analysis of cAMP pathway polymorphisms on DNA from NF1 patients with and without optic pathway glioma, polymorphisms in ADCY8 (AC8) lead to more glioma risk among female patients, although they exhibit a protective effect against glioma in male ones (94). Furthermore, ADCY activity and cAMP levels are higher in benign brain tumors, while the lower levels are associated with a greater degree of malignancy (95). Therefore, there is a good reason to believe that a rise in cAMP will be an important tool in brain tumor treatment.

#### 6.4.8 Cervical Cancer

ADCY8 is the most widely reported subtype implicated in cervical intraepithelial neoplasia. Regarding cervical cancer, miR-181b (a miRNA) promotes the growth of cervical cancer cells and inhibits apoptosis by increasing mRNA degradation and decreasing intracellular cAMP levels, which may be caused by binding to the 30′UTR#2 of AC9 (96). MiR-181b has different roles in tumor cells (97, 98). AC9, which produces cAMP, has been identified as a potential target of miR-181b, which negatively regulates AC9 by reducing AC9 mRNA levels (96). Furthermore, the DNA methylation of *ADCY8* in molecular Pap smears is considered a biomarker of high-grade cervical cytology (99).

#### 6.4.9 Insulinoma

The expression of ADCY1 and calcium channel 2 (CACNA2) greatly enhances insulinoma with a YY1T372R mutation, while they are less expressed in normal β cells (100). The two gene products are involved in the key pathways regulating insulin secretion with the constitutive activation of cAMP and Ca^2+^ signaling pathways implied in insulin secretion (101). Accordingly, the higher expression of ADCY1 and CACNA2D2 may play a crucial role in the pathogenesis of insulinoma.

#### 6.4.10 Prostate Cancer

Significant overexpression of sAC has been detected in prostate cancer. In addition, the inhibition of sAC activity prevents prostate cancer cell proliferation, leading to the release of lactate hydrogenase (LDH), as well as apoptosis. The regulation of sAC-dependent proliferation involves the EPAC/Rap1/B-RAF signaling pathway, and EPAC supports prostate cancer cell proliferation by promoting G2/M phase transition, while PKA has no role (99). The antiproliferative effect of sAC inhibition is seemingly ascribed to the inhibition of MEK1, a downstream target of Rap1/B-Raf signaling, which plays an important role in spindle organization and chromosome stabilization (102), and consequently, can impair spindle formation (103) and cause cell cycle arrest in the G2 phase (104).

## 7 Future Prospects

The ADCY family represents a wide range of functions and plays an important role in a variety of diseases such as neuropathic pain, neurodegenerative diseases, congestive heart failure, asthma, and male contraception. In addition, this family of proteins plays a crucial role in the development of different cancers (26). ADCY isoforms influence the biological behavior of cancer cells such as proliferation, differentiation, apoptosis, and invasive migration, and even have a critical role in cancer cell chemoresistance and sensitivity to radiation *via* cAMP-PKA and other pathways. In recent years, a relationship has been discovered between the family and cancer immunity. The high expression of some ADCY genes activates cancer-associated T-cell function and improves cancer cell immunity (105). This is another mechanism by which ADCYs exert their oncogenic effects, although the exact process is not well understood and should be clarified in continued research. However, better cancer immunity plays an important role in cancer therapy.

Furthermore, the family is expressed at varying levels in cancer cells, some of which are silenced as oncogenes due to DNA methylation. Therefore, a novel cancer treatment approach can involve the restoration of its expression and oncogenic effect by demethylating genes. Similarly, related protein products or specific protein activators can be developed as anticancer drugs to provide more treatment options for patients with various cancers. Currently, available AC agonists like forskolin exhibit some anticancer effects by elevating intracellular cAMP levels. Due to the extensive distribution of ADCYs and the wide range of involved biological functions, no targeted drugs are now available for ADCY isomers, and an urgent need is felt for the appropriate drugs for the various ADCY isomers that truly target cancer therapy.

The identification of a reagent that can be used to treat mutant ADCY indicates great scientific progress and provides important information for future targeted therapy. The present review introduced deleterious hotspot mutants p.E1003K and p.R1116C on ADCY6 as candidates for developing therapeutic strategies targeting cancer cells. The changes in amino acid positioning and H-bond strength can explain how the p.E1003K and p.R1116C mutations affect the complex interaction of ADCY6. Evidently, future studies on protein–protein docking, molecular dynamics, and conformational entropy, as well as principal component analysis, are required to reveal how the two hotspot mutants destroy the structure and function of the complex and increase the fluctuation range. This information presents an excellent basis for understanding the mechanism underlying an enhancement in p-Akt1 activity in carcinogenesis.

## 8 Conclusion

This review highlights the mechanistic functions of the AC family and cAMP in conferring chemoresistance, as well as cancer target therapy, which needs further comprehensive studies for full elucidation. The lower AC and cAMP levels have been reported in many cancers, which promote cancer formation and cancer cell proliferation, reduce apoptosis, and even increase neovascularization and cancer cell migration and invasion. Additionally, deleterious nsSNPs p.E1003K and p.R1116C on ADCY6 are candidates to develop potential therapeutic strategies targeting cancer cells. The possibility of targeting the AC family in oncology opens new opportunities for novel therapeutic strategy development and defines a new approach to improve chemoimmunotherapy efficacy in various cancers. The importance of immunotherapy and targeted therapies has become growing in cancer treatment, and the anticancer therapies targeting the ADCY family, certainly ADCY1 and ADCY6, are becoming increasingly possible. Furthermore studies are required to elucidate the paradoxical effects of the other members of the family pathway in response to cancer therapy. In particular, further investigations should be conducted to clarify the interplay between the overexpression of ADCY signaling and cAMP-dependent genes in various tumors. Therefore, targeting the different isoforms of the ADCY family may be a new strategy for treating various cancers.

## Author Contributions

RG and TL checked the relevant literature and wrote the original draft of the manuscript. MD and XW performed the data interpretation and visualization in the form of diagrams and tables. SI and QW performed the project administration and funding acquisition. All authors have read and agreed to the published version of the manuscript and agreed to be accountable for all aspects of the work.

## Funding

This research was funded by Southwest Medical University (SWMU), grant number HR21013.

## Conflict of Interest

The authors declare that the research was conducted in the absence of any commercial or financial relationships that could be construed as a potential conflict of interest.

## Publisher’s Note

All claims expressed in this article are solely those of the authors and do not necessarily represent those of their affiliated organizations, or those of the publisher, the editors and the reviewers. Any product that may be evaluated in this article, or claim that may be made by its manufacturer, is not guaranteed or endorsed by the publisher.
